# MRI radiomics-based predictive modeling for risk stratification and prognostication in oral tongue carcinoma

**DOI:** 10.3389/fonc.2026.1901400

**Published:** 2026-09-03

**Authors:** Nivedita Chakrabarty, Swapnil U. Rane, Umesh Sherkhane, Sourabh More, Nitin Shetty, Sarbani Ghosh Laskar, Arjun Singh, Vanita Noronha, Kumar Prabhash, Pankaj Chaturvedi

**Affiliations:** 1 Department of Radiodiagnosis, Tata Memorial Centre, Homi Bhabha National Institute (HBNI), Mumbai, India; 2 Advanced Centre for Treatment Research and Education in Cancer, Navi Mumbai, India; 3 Department of Pathology, Tata Memorial Centre, Homi Bhabha National Institute (HBNI), Mumbai, India; 4 Department of Artificial Intelligence and Machine Learning, Tata Memorial Centre, Homi Bhabha National Institute (HBNI), Mumbai, India; 5 Department of Radiation Oncology, Tata Memorial Centre, Homi Bhabha National Institute (HBNI), Mumbai, India; 6 Department of Surgical Oncology, Tata Memorial Centre, Homi Bhabha National Institute (HBNI), Mumbai, India; 7 Department of Medical Oncology, Tata Memorial Centre, Homi Bhabha National Institute (HBNI), Mumbai, India

**Keywords:** MRI-based radiomics, oral tongue cancer, predictive modeling, prognostic modeling, risk stratification

## Abstract

**Introduction:**

This study aims to predict recurrence-free survival (RFS) and overall survival (OS), to stratify risk using radiomics, radiology semantic, clinical, and pathology models—individually and in combination—derived from pre-treatment magnetic resonance imaging (MRI), post-operative histopathology, age, and adjuvant therapy details in oral tongue squamous cell carcinoma (OTSCC), and to correlate National Comprehensive Cancer Network (NCCN) low + intermediate- and high-risk groups with the risk stratification provided by the radiomics models. The secondary objectives included predicting post-operative perineural invasion (PNI), metastatic lymph nodes, and pathological extranodal extension (pENE).

**Methods:**

This retrospective cohort study included 219 treatment-naive OTSCC patients who underwent pre-treatment MRI (January 2021–December 2022) with 2-year follow-up. Radiomics features were extracted from T1-weighted, T2-weighted, and contrast-enhanced T1-weighted (T1c) sequences. Lasso–Cox and DeepSurv models were evaluated for RFS and OS prediction, while random forest and logistic regression models were assessed for PNI, metastatic lymph nodes, and pENE prediction. Radiomics, radiology semantic, clinical, and pathology models were evaluated individually and in combination. Risk stratification was evaluated using the log-rank test.

**Results:**

Among 219 patients (mean age 46.99 ± 10.94 years), Lasso–Cox outperformed DeepSurv for RFS and OS prediction. The radiomics combined model (T1 + T2 + T1c radiomics + radiology semantic + clinical + pathology) achieved the best performance for RFS and non-inferior performance to the pathology model for OS prediction. The pre-treatment radiomics models effectively stratified the patients into high- and low-risk groups for recurrence and OS and also identified low-risk patients within the NCCN high-risk group and high-risk patients within the NCCN low + intermediate-risk group for recurrence. The T1c radiomics model demonstrated the best overall performance for predicting metastatic lymph nodes. The radiology semantic features model demonstrated the highest predictive performance for PNI and pENE.

**Conclusions:**

The radiomics combined and pre-treatment radiomics models demonstrate potential for prognostication and risk-adapted management. By identifying low-risk patients within the NCCN high-risk group and high-risk patients within the NCCN low + intermediate-risk group for recurrence, these models may support treatment de-escalation and escalation, respectively, pending prospective multicenter validation. Furthermore, prediction of metastatic lymph nodes, PNI, and pENE from pre-treatment radiomics models may inform decisions regarding elective neck dissection and guide adjuvant therapy following prospective validation.

## Introduction

Radiomics is an emerging field in head and neck oncology that enables the non-invasive extraction of quantitative imaging features, such as texture, shape, and intensity, from standard modalities like magnetic resonance imaging (MRI), revealing patterns beyond visual assessment and aiding clinical decision-making (1–5). Oral tongue squamous cell carcinoma (OTSCC), the most common oral cavity cancer subtype, ranks as the 16th most common cancer worldwide (6). MRI is the preferred imaging modality for OTSCC because of its superior soft tissue contrast (7–9). Surgery remains the mainstay of treatment; however, even early-stage disease carries a risk of locoregional recurrence, adversely affecting overall survival (OS) (10, 11). Recurrence rates range from 10%–25% in early-stage disease to 40%–60% in advanced stages (12, 13). Borderline resectable tumors may receive neoadjuvant chemotherapy (NACT), although only 30%–40% respond, with no reliable pre-treatment predictor currently available (14, 15). In unresectable OTSCC, treatment options include definitive chemoradiotherapy (CRT), palliative therapy, or supportive care (10, 16).

Extranodal extension (ENE), unless overtly evident on imaging, and perineural invasion (PNI) are established adverse prognostic factors in OTSCC but can only be definitively identified on post-operative histopathology (17–21). Similarly, worst pattern of invasion (WPOI) scores 4 and 5 are associated with poorer OS, higher recurrence, and increased nodal metastasis, yet they also rely on post-operative histopathology (22, 23). Recent MRI-based studies have additionally identified adverse pre-treatment imaging biomarkers, including peritumoral edema, tumor ulceration, peritumoral hyperenhancement (particularly when >2.65 mm), and tumor proximity within 5 mm of the hyoid bone (24).

In unresectable OTSCC, imaging remains the only modality for identifying adverse prognostic features before treatment. Recognizing high-risk markers on pre-treatment MRI may facilitate treatment stratification by enabling aggressive therapy in early-stage high-risk cases, guiding adjuvant treatment planning in both resectable and unresectable disease, and informing decisions regarding elective neck dissection and personalized follow-up strategies based on risk assessment, which form the basis of our study.

Although several artificial-intelligence-based studies have attempted to predict survival outcomes in head and neck cancers, none have incorporated radiological semantic features into their models nor have any predicted PNI in OTSCC using baseline MRI (25–28). In addition, to the best of our knowledge, no previous study has investigated the correlation between clinicopathological low + intermediate- and high-risk groups, as defined by the National Comprehensive Cancer Network (NCCN) recommendations, and the low- and high-risk stratification generated by a radiomics model.

The primary objectives of our study were to predict recurrence-free survival (RFS) and OS using radiomics model (using treatment naive MRI of OTSCC), radiology semantic features model (using treatment naive MRI of OTSCC), pathology model (using post-operative histopathological features), clinical model (using age and adjuvant therapy details), and combined radiomics–radiology semantic features–pathology–clinical model (radiomics combined model) and to correlate clinico-pathologic low + intermediate- and high risk groups (based on NCCN recommendations) with the risk stratification (low and high risk) provided by the radiomics models.

The secondary objective was to predict PNI, metastatic nodes, and pathological ENE (pENE) from primary tumor using radiomics models, radiology semantic features model, and combined radiomics and radiology semantic features model on treatment-naive MRI of OTSCC.

## Methods

### Study design and study population

A retrospective cohort study was conducted at a tertiary cancer center. Treatment-naive MRI scans of upfront operated OTSCC patients from January 1, 2021 to December 31, 2022 were reviewed after the approval of the Institutional Ethics Committee. A total of 219 patients meeting the inclusion and exclusion criteria were included. A dedicated head and neck radiologist with over 12 years of experience assessed the MRI studies and recorded relevant semantic features. Post-operative histopathological findings and 2-year follow-up data were retrieved from hospital electronic medical records (EMR).

The inclusion criteria required that a pre-treatment MRI be performed within 4 weeks prior to upfront surgery for OTSCC. Eligible cases were expected to have T1-weighted imaging (T1WI), T2WI, short tau inversion recovery (STIR), thin-section (≤2 mm) contrast-enhanced fast spoiled gradient echo (CE-FSPGR), and 3-mm-section-thickness CE T1WI (T1c) available in the picture archiving and communication system (PACS). Additionally, a post-operative histopathology report (HPR) and details of adjuvant treatment were required to be available on EMR. Cases were excluded if any MRI sequences were missing, if treatment-naive MRI images were not available, or if the tumor epicenter was located outside the oral tongue. The MRI protocol is outlined in **Supplementary Table 1**.

**Figure 1:**
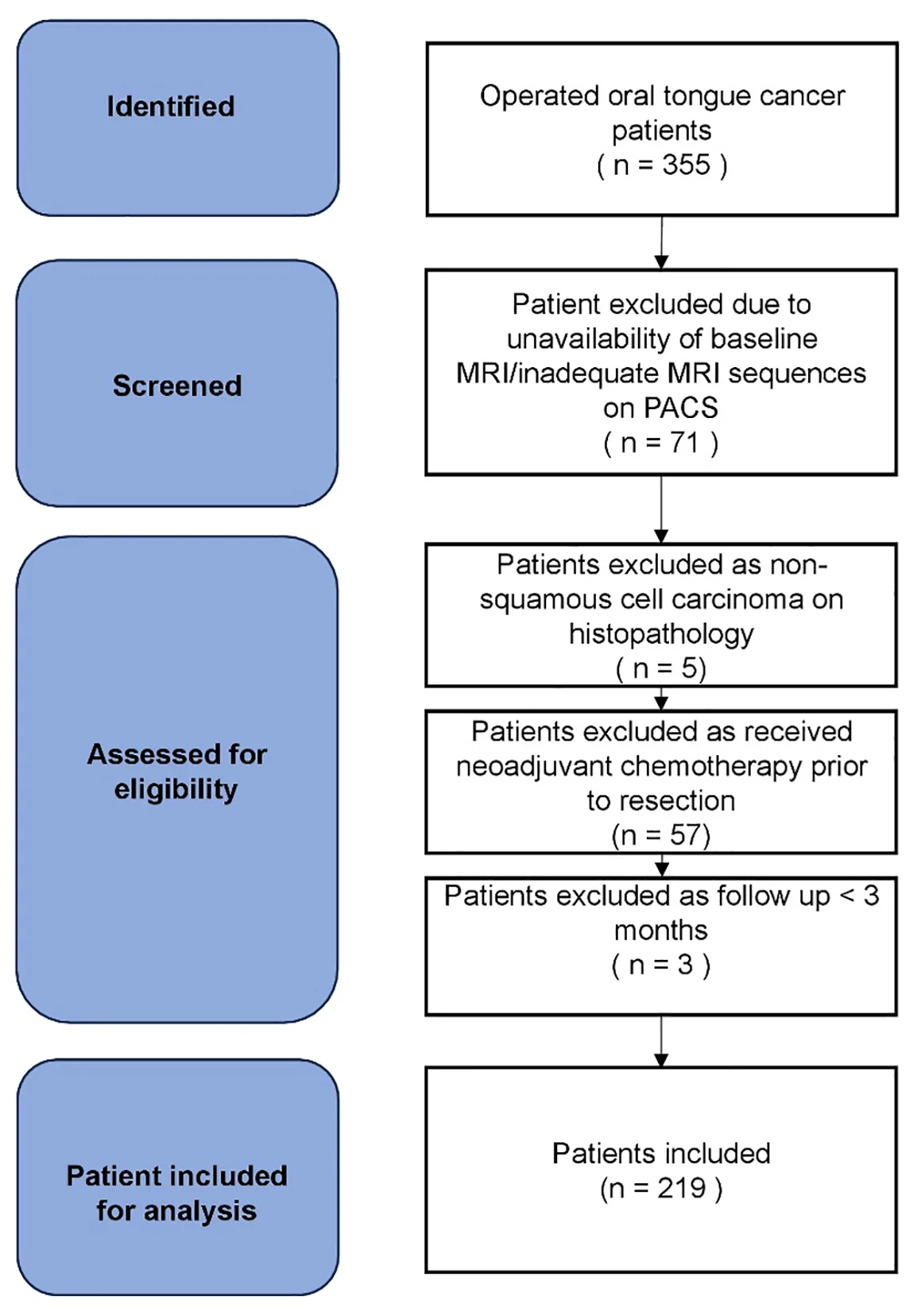
Flow diagram for the selection of eligible patients.

**Figure 1** shows the flow diagram for the selection of eligible patients.

**Table 1** summarizes the radiologic semantic features, clinical variables, and post-operative pathological findings used in developing the predictive models for RFS and OS. For the fulfilment of the secondary objective, radiological T category was also used in addition to the radiology semantic features detailed in **Table 1**.

**Table 1:** Radiologic semantic features, clinical variables, and post-operative pathological findings employed in constructing the predictive model.

Section	Variable	Documentation
Radiology semantic features (on baseline/treatment naïve MRI)	Distance of tumor from the lingual septum	>5 mm/≤5 mm/bulging of the lingual septum to contralateral side/tumor frankly crossing to the contralateral side
	Imaging inner tumor margin	Lobulated/irregular/ill-defined, infiltrative
	Peritumoral hyperenhancement (PH) on CE FSPGR sequence	Present/absent
	PH width (maximum width measured if PH is present)	Expressed in millimeters
	Peritumoral edema (PE) on STIR (observed as faint homogeneous hyperintensity beyond tumor margins)	Present/absent
	Tumor ulceration (TU)	Present/absent
	Extension to base of tongue	Present/absent
	Distance from hyoid bone	≤10 mm/>10 mm
	Extension to <5 mm from hyoid bone	Present/absent
Clinical data	Age	Patient’s age in years
	Adjuvant treatment	No adjuvant/adjuvant CRT/adjuvant RT
Post-operative pathology data (from HPR available on EMR)	pT category	pT1/pT2/pT3/pT4a/pT4b
	pN category	pN0/pN1/pN2a/pN2b/pN2c/pN3a/pN3b
	Perineural invasion (PNI)	Present/absent
	Pathological extranodal extension (pENE)	Present/absent
	Worst pattern of invasion (WPOI)	WPOI 1–5

CRT, chemoradiotherapy; RT, radiotherapy; pT, pathological primary tumor; pN, pathological regional node; HPR, histopathology report; EMR, electronic medical records; STIR, Short tau inversion recovery.

### Clinico-pathologic risk stratification

Clinico-pathologic risk stratification of the study population into low-, intermediate-, and high-risk groups were done based on the NCCN recommendations of adjuvant therapy for oral cavity cancers (11).

### Image segmentation and radiomics feature extraction

**Figure 2:**
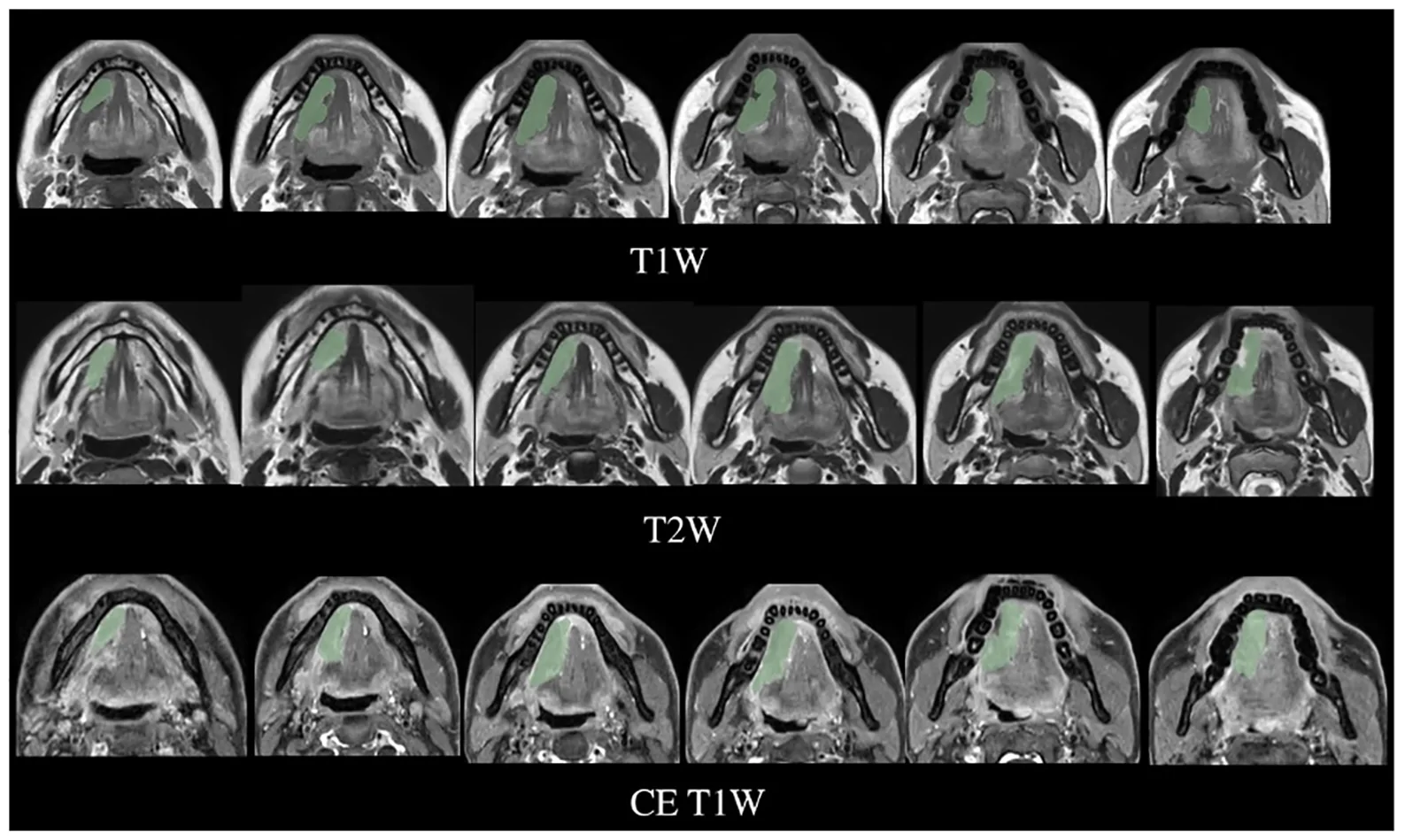
Separate manual segmentation masks on T1W (green mask in first row), T2W (green mask in second row), and T1cW (green mask in third row) MRI sequences for the same patient with right lateral border oral tongue squamous cell carcinoma.

Segmentation of the primary tumor was performed separately on T1, T2, and T1c baseline MRI sequences in patients with biopsy-proven OTSCC (**Figure 2**). Initial segmentation was done by a radiology technician using 3D Slicer, and all segmentations were subsequently reviewed and either confirmed or modified by a dedicated head and neck radiologist with over 12 years of experience. MRI images were acquired either in General Electric (GE)-Signa explorer 1.5T or Philips-Ingenia 1.5T. Compatible Pyradiomics software was used to extract first-order statistics, shape-based features, and texture features [gray-level co-occurrence matrix (GLCM), gray-level run length matrix (GLRLM), gray-level size zone matrix (GLSZM), and neighborhood gray tone difference matrix (NGTDM)] from each of the sequences for every tumor. A bin width of 9 and a voxel size of 0.7 × 0.7 × 2.0 mm were used for the radiomics feature extraction.

### Follow-up

Follow-up data for 2 years (until 31/12/2024) were collected from the EMR for the calculation of RFS (local, regional, or distant recurrence) and OS from the date of surgery.

### Protocol for post-treatment follow-up and recurrence assessment

At our institution, patients are followed clinically every 3 months for the first 2 years after completing treatment and then every 6 months for the subsequent 3 years. After 5 years, follow-up visits are conducted annually. The selection of imaging—either MRI (using the same protocol as the baseline, as outlined in **Supplementary Table 1**) or fluorodeoxyglucose positron emission tomography (FDG-PET) with contrast-enhanced computed tomography (CECT)—is based on clinical suspicion and examination findings. Biopsy is performed when necessary to confirm recurrence.

### Statistical analysis

OS was defined as the time from the date of surgery to the date of death from any cause or last follow-up, expressed in months. RFS was defined as the time from the date of surgery to the date of first documented recurrence (local, regional, or distant). All time-to-event outcomes were calculated in months as the difference between the event date and the date of surgery. Patients without events were censored at the last follow-up date. Descriptive analyses were conducted for demographic data such as sex, while continuous variables such as age were expressed as the mean ± standard deviation or as median (interquartile range [IQR]), depending on the normality of the data. Kaplan–Meier (KM) survival curves were generated to compare RFS among risk groups, and statistical significance was assessed using the log-rank test. KM survival analyses were performed to compare RFS among the four NCCN-radiomics risk groups (agreement low, upstaged, downstaged, and agreement high). Statistical significance between survival curves was assessed using the global log-rank test. Pairwise log-rank tests were additionally performed for specific comparisons of interest.

**Figure 3:**
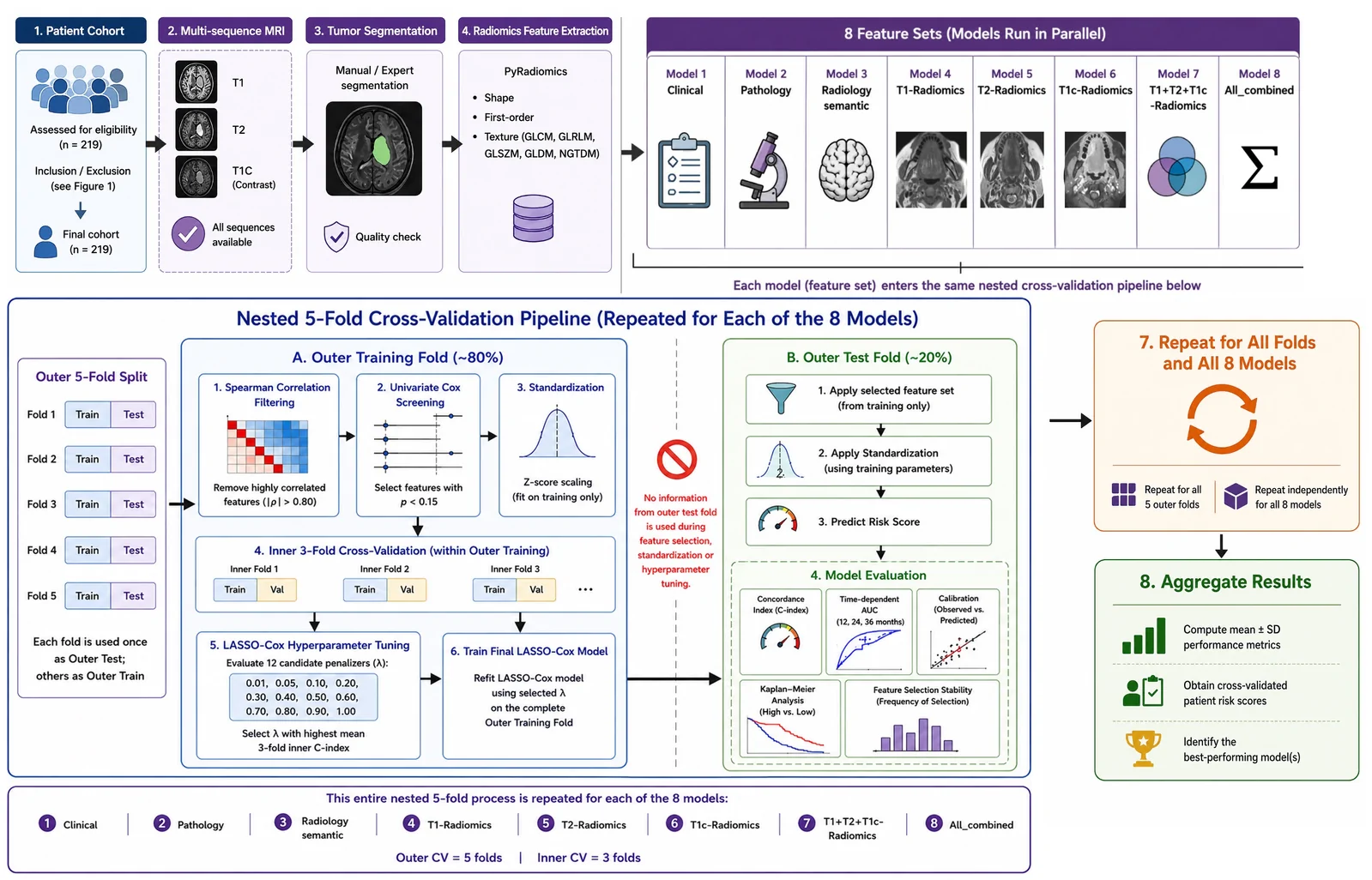
Workflow diagram mapping the computational pipeline end to end.

An overview of the complete modeling workflow is illustrated in **Figure 3**. Briefly, radiomics, clinical, pathological, and radiology semantic features were extracted and subsequently analyzed using a leakage-free nested cross-validation framework. Feature selection, standardization, hyperparameter optimization, model training, and performance evaluation were performed exclusively within the training data of each outer fold to avoid information leakage and obtain unbiased estimates of model performance.

## Prediction of RFS and OS

### Feature selection

A total of 107 radiomic features were extracted from each MRI sequence, resulting in 321 combined radiomic features for the integrated model. To avoid information leakage, all feature selection procedures were performed exclusively within the training data of each outer cross-validation fold.

Initially, highly correlated features were removed using Spearman correlation analysis (threshold >0.8) applied only to the outer training cohort. Subsequently, the remaining features underwent univariate Cox proportional hazards analysis, and features demonstrating a significance level of *p* < 0.15 were retained for further modeling. A liberal screening threshold was adopted to preserve potentially informative prognostic variables, consistent with previous recommendations advocating thresholds between 0.15 and 0.25 during initial feature selection stages (29). Features selected within each training fold were then carried forward for model development. Feature selection stability was assessed by recording the frequency with which variables were retained across the five outer folds.

### Regularized (Lasso) Cox proportional hazards model with nested cross-validation

The prognostic performance for RFS and OS was evaluated using a leakage-free nested cross-validation framework based on an L1-regularized Cox proportional hazards model (Lasso–Cox). RFS was defined as the time from diagnosis to local, regional, or distant recurrence, with recurrence status represented by the binary variable “Disease_Recurrence”. OS was similarly defined as time to death from any cause or last follow-up.

The nested cross-validation framework consisted of a fivefold outer loop and a threefold inner loop. In each outer iteration, approximately 80% of patients were allocated to the training cohort, and the remaining 20% constituted an independent test cohort. Importantly, all preprocessing steps, including feature selection, standardization, and hyperparameter optimization, were performed exclusively within the outer training cohort, while the outer test fold remained completely unseen until final evaluation.

Within each outer training fold, the following sequential steps were performed:

1. Spearman correlation filtering (threshold > 0.8)
2. Univariate Cox feature selection (*p* < 0.15)
3. Feature standardization using parameters derived solely from the training data
4. Hyperparameter optimization using inner cross-validation

This process was repeated across all five outer folds, ensuring that each patient served once as independent test data.

To assess the adequacy of data partitioning, baseline clinical and pathological characteristics between training and test cohorts were compared within each outer fold. Continuous variables were analyzed using Student’s *t*-tests, whereas categorical variables were evaluated using chi-square tests. No major differences were observed between training and test cohorts, indicating reasonably balanced data partitions across the nested cross-validation framework.

### Modeling procedure for RFS and OS prediction

Model optimization was performed by tuning the L1 regularization parameter (penalizer) across 12 candidate values (0.01, 0.05, 0.10, 0.20, 0.30, 0.40, 0.50, 0.60, 0.70, 0.80, 0.90, and 1.00). For each candidate penalizer, an L1-regularized Cox model was trained within the inner threefold cross-validation loop and evaluated using the concordance index (C-index). The penalizer yielding the highest mean validation C-index across the inner folds was selected as the optimal parameter.

A final Lasso–Cox model using the selected penalizer was subsequently trained on the complete outer training cohort and evaluated on the independent outer test fold. Patient-specific risk scores were generated using predicted log partial hazards, and non-zero model coefficients were recorded to identify prognostically informative variables.

### Performance metrics

Model performance was primarily assessed using the C-index. Training C-indices were calculated within the outer training cohorts, while test C-indices were obtained from the independent outer test cohorts to estimate generalizable predictive performance.

Cross-validated patient-level risk scores were aggregated across all outer folds to ensure that each patient received a prediction generated from a model that had not been trained using that patient’s data. Time-dependent receiver operating characteristic (ROC) analyses were performed at 12, 24, and 36 months, and corresponding time-dependent AUC values were calculated. Calibration curves were additionally generated to evaluate the agreement between predicted and observed survival probabilities.

Eight predictive models were developed and compared, namely:

1. Clinical model
2. Pathological model
3. Radiology semantic features model
4. T1 radiomics model
5. T2 radiomics model
6. T1c radiomics model
7. Combined T1 + T2 + T1c radiomics model
8. Fully integrated model combining clinical, pathological, semantic, and radiomics features

All models were developed using the identical leakage-free nested cross-validation framework to enable a fair comparison.

### Risk stratification and Kaplan–Meier analysis

Patient-specific cross-validated risk scores obtained from the nested Cox models were dichotomized into low- and high-risk groups using the median risk score as the cutoff. The median threshold was selected to provide an unbiased and reproducible risk stratification approach independent of outcome information and to minimize overfitting associated with optimal cut-point methods.

Kaplan–Meier survival curves were subsequently generated to compare the survival outcomes between risk groups. Statistical significance was assessed using the log-rank test, and hazard ratios (HRs) with corresponding 95% confidence intervals (CI) were estimated using Cox proportional hazards regression.

Survival analyses included KM curves, numbers-at-risk tables, log-rank *p*-values, hazard ratios, and corresponding 95% CI.

Additionally, patients were stratified according to pathological stage into the following:

1. Early-stage disease (pT1/T2 N0)
2. Locally advanced disease (all remaining patients)

The prognostic value of conventional staging criteria was similarly evaluated using KM analysis, Cox regression, and log-rank testing.

A deep-learning-based survival model (DeepSurv) was also trained and tested to predict RFS and OS.

## Prediction of PNI, metastatic nodal involvement, and ENE

### Feature selection

The dataset was initially divided into stratified training (70%) and independent test (30%) cohorts while preserving the distribution of outcome classes. To prevent information leakage, all preprocessing, feature selection, and model optimization procedures were performed exclusively within the training cohort.

First, Spearman correlation analysis (threshold > 0.8) was applied only to the training data to identify and remove highly correlated radiomics features. The same retained feature subset was subsequently applied to the independent test cohort without recalculating correlations.

Within the training cohort, feature selection was further performed using stratified fivefold cross-validation and recursive feature elimination with cross-validation (RFECV), with receiver operating characteristic area under the curve (ROC-AUC) as the optimization metric. Features selected in each fold were recorded, and feature selection stability was assessed based on selection frequency across folds. The top 20 most frequently selected features were subsequently retained for final model development.

No information from the independent test cohort was used during correlation filtering, feature selection, hyperparameter tuning, threshold optimization, or model training. All feature selection procedures were performed exclusively within the training cohort to avoid information leakage.

### Model development

Six independent datasets were used for model development, namely:

1. T1 radiomics features
2. T2 radiomics features
3. T1c radiomics features
4. T1 + T2 + T1c radiomics features
5. Radiology semantic features
6. Integrated radiomics and radiology semantic features

For each dataset, both logistic regression (LR) and random forest (RF) classifiers were developed.

Hyperparameter optimization was performed exclusively within the training cohort using nested stratified cross-validation and GridSearchCV. Following optimization, final models were retrained using the complete training cohort and subsequently evaluated on the independent 30% test cohort.

To improve classification performance and account for class imbalance, the optimal probability threshold was determined using the Youden index derived from the training ROC curve rather than using a fixed threshold of 0.5.

### Performance evaluation

Model performance was evaluated on the independent test cohort using multiple discrimination and classification metrics.

### Area under the ROC curve

Discriminative performance was primarily assessed using the ROC-AUC. ROC curves were generated by plotting sensitivity against 1 − specificity across varying probability thresholds. 95% CI for AUC values was estimated using bootstrap resampling with 2,000 iterations.

### Accuracy

Accuracy was calculated as follows:


$$\text{Accuracy}=\frac{\text{TP}+\text{TN}}{\text{TP}+\text{TN}+\text{FP}+\text{FN}}$$

where TP, TN, FP, and FN denote true positives, true negatives, false positives, and false negatives, respectively.

### Sensitivity (recall)

Sensitivity was calculated as follows:


$$\text{Sensitivity}=\frac{\text{TP}}{\text{TP}+\text{FN}}$$

representing the proportion of positive cases correctly identified by the model.

### Specificity

Specificity was calculated as follows:


$$\text{Specificity}=\frac{\text{TN}}{\text{TN}+\text{FP}}$$

representing the proportion of negative cases correctly identified.

### Positive predictive value


$$\text{PPV}=\frac{\text{TP}}{\text{TP}+\text{FP}}$$

representing the probability that a predicted positive case truly belongs to the positive class.

### Negative predictive value


$$\text{NPV}=\frac{\text{TN}}{\text{TN}+\text{FN}}$$

representing the probability that a predicted negative case truly belongs to the negative class.

### F1-score

The F1-score was calculated as the harmonic mean of precision and recall:


$$\text{F}1=2\times \frac{\text{Precision}\times \text{recall}}{\text{Precision}+\text{recall}}$$

### Balanced accuracy

Balanced accuracy was calculated, as follows, to account for class imbalance:


$$\text{Balanced accuracy}=\frac{\text{Sensitivity}+\text{specificity}}{2}$$

All performance metrics were reported on the independent test cohort.

### Receiver operating characteristic analysis

Receiver operating characteristic curves were generated for both LR and RF models using predicted probabilities obtained from the independent test cohort.

Mean ROC curves and corresponding AUC values with 95% CI were reported for all predictive models.

### Calibration analysis

Model calibration was evaluated to assess the agreement between predicted probabilities and observed event frequencies. Calibration curves were generated using the independent test cohort by dividing predicted probabilities into quantile-based bins and comparing mean predicted probabilities with observed event proportions within each bin.

A perfectly calibrated model would demonstrate predictions lying along the 45° reference line. Calibration analysis was performed for both LR and RF models.

### Decision curve analysis

Decision curve analysis (DCA) was performed to evaluate the potential clinical utility of the developed models.

Net benefit was calculated across a range of threshold probabilities according to the following:


$$\text{Net benefit}=\frac{\text{TP}}{N}-\frac{\text{FP}}{N}\times \frac{p_{t}}{1-p_{t}}$$

where $p_{t}$ denotes the threshold probability and N represents the total number of patients.

Decision curves were compared against two default clinical strategies:

1. Treat-all strategy
2. Treat-none strategy

Models demonstrating higher net benefit than both default strategies across clinically relevant threshold probabilities were considered to possess potential clinical utility.

### Model explainability

To improve model transparency and interpretability, SHAP (SHapley Additive exPlanations) analyses were performed. For RF models, SHAP values were estimated using TreeExplainer, whereas LinearExplainer was used for LR models.

SHAP analyses were performed using the final optimized models after completion of feature selection and hyperparameter tuning. Therefore, the displayed SHAP plots represent feature contributions derived from the final fitted models and were not generated independently within cross-validation folds.

Feature importance was quantified using mean absolute SHAP values and visualized using SHAP summary plots and beeswarm plots.

This approach provided a patient-level interpretation of model predictions and facilitated the identification of potential imaging biomarkers associated with the following:

1. PNI
2. Metastatic nodal involvement
3. pENE

RF feature importance analyses were additionally performed to complement SHAP-based explainability.

**Supplementary Table 2** shows feature selection stability.

## Results

### Demographic characteristics

There were 179 male and 40 female patients in our study. The mean and median age of the patients were 46.99 ± 10.94 standard deviation (SD) years and 46 years (interquartile range [IQR]: 39–54.50), respectively. Median follow-up was 28.13 months.

Median RFS was 27.7 months. **Supplementary Table 3** shows the detailed stage-wise distribution of the study population along with the details of adjuvant treatment received. There was no statistically significant difference in age (*p*-value 0.6570) across the three groups (RT vs. CRT vs. no adjuvant), which means that age is relatively well balanced among them.

### Predictive modeling for RFS and risk stratification

The combined model incorporating T1 + T2 + T1c radiomics + radiology semantic features + clinical features + pathology features (radiomics combined model) outperformed individual models with a mean train concordance (C) index of 0.75 ± 0.04 SD and mean test C index of 0.70 ± 0.11 SD for predicting RFS. The T1 + T2 + T1c radiomics model showed mean train and test C indices of 0.67 ± 0.02 SD and 0.62 ± 0.07 SD, respectively. The T1c radiomics model showed better performance compared to either T1 or T2 radiomics model, with mean train and test C indices of 0.64 ± 0.01 SD and 0.63 ± 0.04 SD, respectively. Among individual models, the pathology-only model outperformed all of the other individual models, showing mean train and test C indices of 0.69 ± 0.01 SD and 0.68 ± 0.05 SD, respectively. The time-dependent AUC and calibration curves for each of the models for RFS prediction are shown in **Supplementary Figures 1–8**.

**Figure 4:**
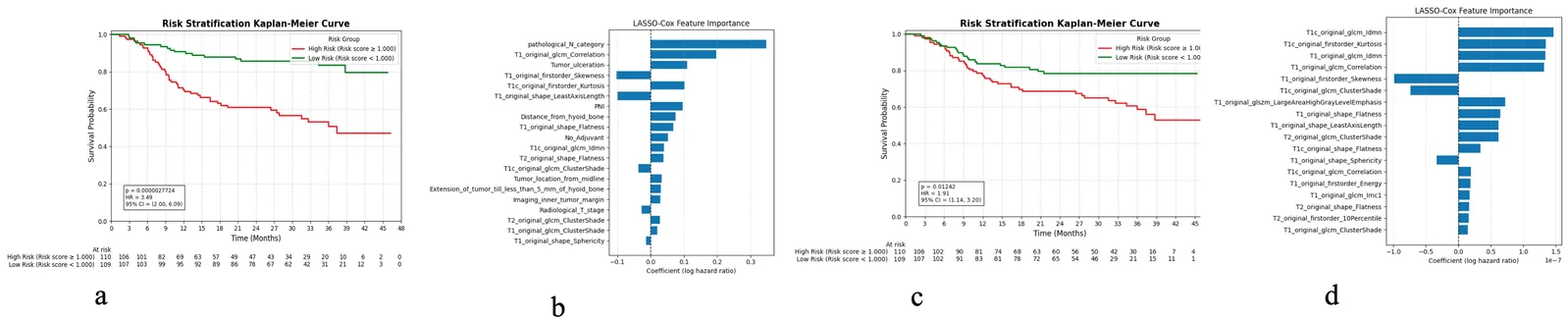
**(a–d)** Risk stratification and important features for the prediction of recurrence-free survival. **(a, b)** High-risk (≥1) and low-risk (<1) groups **(a)** and important features **(b)** of the radiomics combined model (T1 + T2 + T1c radiomics + radiology semantic features + clinical + pathology). **(c, d)** High-risk (≥1) and low-risk (<1) groups **(c)** and important features **(d)** of the T1 + T2 + T1c radiomics model.

**Figure 5:**
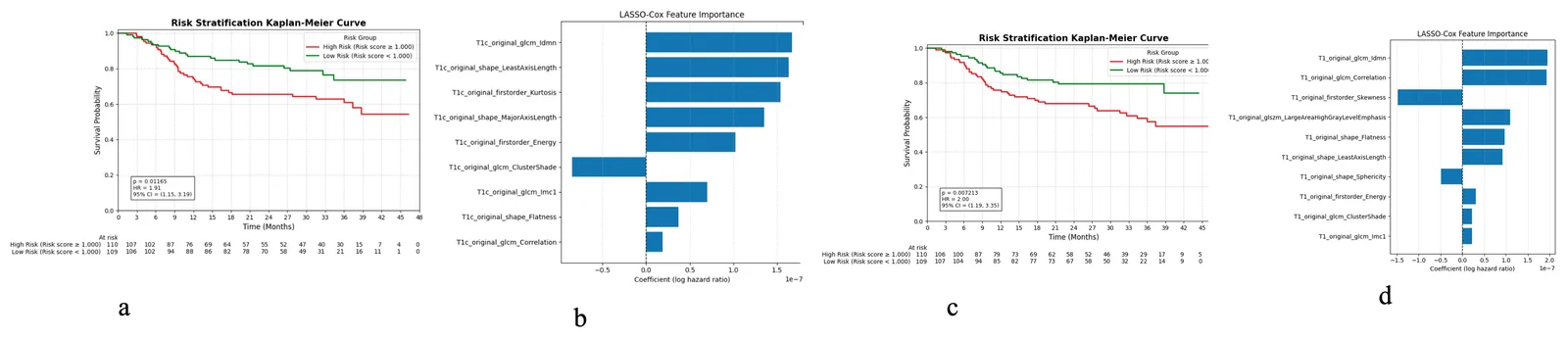
**(a–d)** Risk stratification and important features for the prediction of recurrence-free survival. **(a, b)** High-risk (≥1) and low-risk (<1) groups **(a)** and important features **(b)** of the T1c radiomics model. **(c, d)** High-risk (≥1) and low-risk (<1) groups **(c)** and important features **(d)** of the T1 radiomics model.

Significant *p*-values for predicting high- versus low-risk groups were seen with the radiomics combined model (*p*-value of 0.0000027724), T1 + T2 + T1c radiomics model (*p*-value of 0.01242), T1c radiomics model (*p*-value of 0.01165), and T1 radiomics model (*p*-value of 0.007213). The risk groups and important features of the radiomics combined model and T1W + T2W + T1c radiomics model for RFS prediction are depicted in **Figure 4**. The risk groups and important features of the T1c and T1 radiomics models are shown in **Figure 5**.

Important features in the rest of the models for RFS prediction are included in **Supplementary Figures 9–12**.

Lasso–Cox was found to be superior than DeepSurv for radiomics combined as well as individual RFS predictive modeling.

### Predictive modeling for OS and risk stratification

The performance of radiomics combined model incorporating T1 + T2 + T1c radiomics + radiology semantic features + clinical features + pathology features showed a mean train C index of 0.73 ± 0.03 SD and a mean test C index of 0.69 ± 0.07 SD, which were non-inferior to the pathology model (mean train C index of 0.72 ± 0.02 SD and mean test C index of 0.71 ± 0.07 SD) for predicting OS. The T1 + T2 + T1c radiomics model demonstrated a mean train C index of 0.65 ± 0.02 SD and a mean test C index of 0.59 ± 0.07 SD, and the T1c radiomics model was better than either T1 or T2 radiomics model with mean train and test C indices of 0.65 ± 0.02 SD and 0.64 ± 0.06 SD, respectively. The time-dependent AUC and calibration curves for each of the models for OS prediction are shown in **Supplementary Figures 13–20**.

Significant *p*-values for predicting high- versus low-risk groups were seen with the radiomics combined model (*p*-value of 0.0000008346), T1c radiomics model (*p*-value 0.004144), and T1 radiomics (*p*-value of 0.04862).

**Figure 6:**
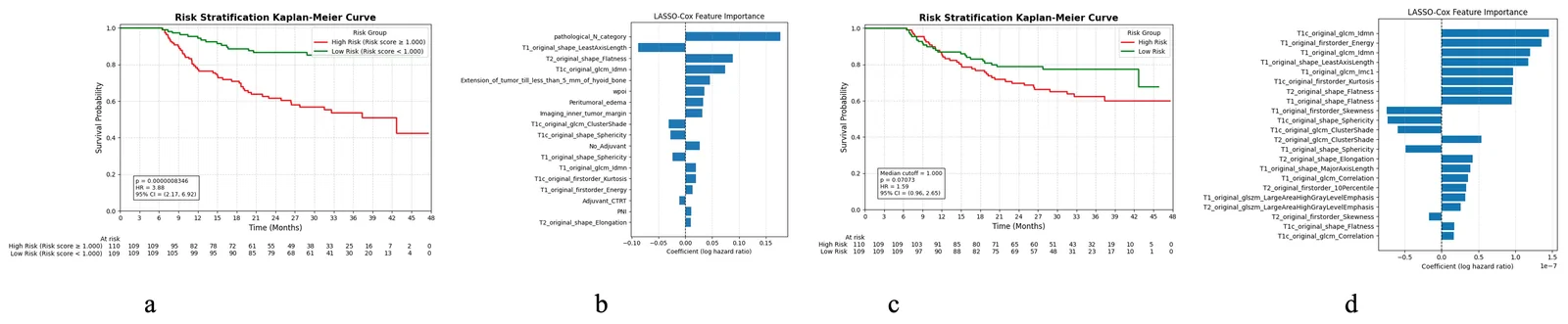
**(a–d)** Risk stratification and important features for the prediction of overall survival. **(a, b)** High-risk (≥1) and low-risk (<1) groups **(a)** and important features **(b)** of the radiomics combined model (T1 + T2 + T1c radiomics + radiology semantic features + clinical + pathology). **(c, d)** High-risk (≥1) and low-risk (<1) groups **(c)** and important features **(d)** of the T1 + T2 + T1c radiomics model.

**Figure 7:**
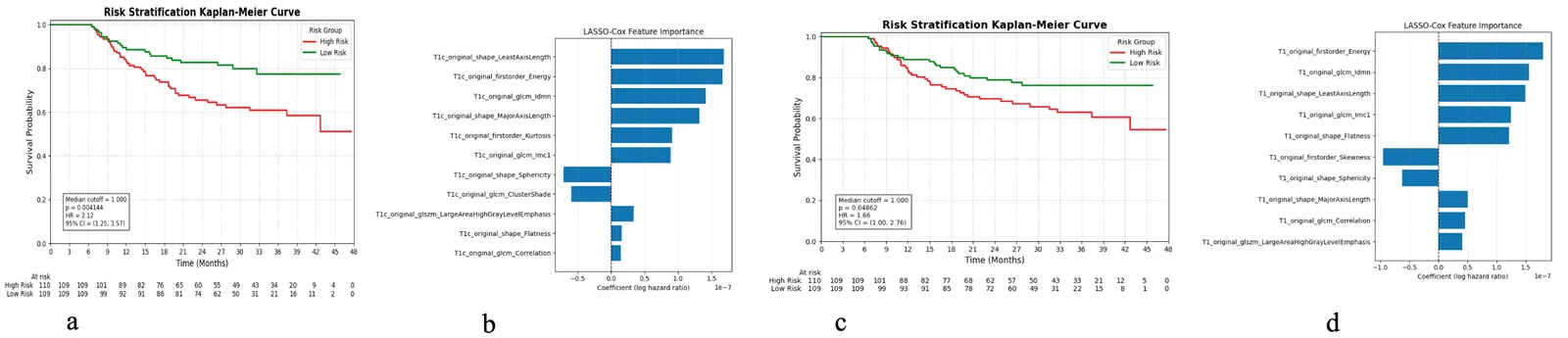
**(a–d)** Risk stratification and important features for the prediction of overall survival. **(a, b)** High-risk (≥1) and low-risk (<1) groups **(a)** and important features **(b)** of the T1c radiomics model. **(c, d)** High-risk (≥1) and low-risk (<1) groups **(c)** and important features **(d)** of the T1 radiomics model.

The risk groups and important features of the radiomics combined model and T1W + T2W + T1c radiomics model for OS prediction are depicted in **Figure 6**. The risk groups and important features in the T1c and T1 radiomics models for OS prediction are shown in **Figure 7**.

Important features in the rest of the models for OS prediction are included in **Supplementary Figures 21–24**.

Lasso–Cox was found to be superior than DeepSurv for radiomics combined as well as individual OS predictive modeling.

### Predictive modeling for PNI

PNI was found in 89 patients (40.63%). The RF model outperformed the LR model in predicting PNI across both the combined and individual models, except for the T2 radiomics model in which the LR model demonstrated superior performance. The radiology semantic features RF model demonstrated the highest predictive performance among all models, achieving mean AUC, accuracy, sensitivity, specificity, PPV, NPV, F1-score, and balanced accuracy of 0.730 (95% CI: 0.588–0.857), 0.714 (95% CI: 0.606–0.818), 0.668 (95% CI: 0.481–0.846), 0.746 (95% CI: 0.605–0.878), 0.646 (95% CI: 0.467–0.821), 0.763 (95% CI: 0.629–0.895), 0.652 (95% CI: 0.500–0.794), and 0.707 (95% CI: 0.592–0.819), respectively. Among the radiomics-based models, T2 radiomics LR model achieved the highest mean AUC of 0.72 (95% CI: 0.578–0.841) for predicting PNI. However, the T1 radiomics RF model demonstrated the best overall predictive performance, with a mean AUC, accuracy, sensitivity, specificity, PPV, NPV, F1-score, and balanced accuracy of 0.710 (95% CI: 0.583–0.831), 0.680 (95% CI: 0.576–0.788), 0.517 (95% CI: 0.333–0.704), 0.793 (95% CI: 0.659–0.911), 0.635 (95% CI: 0.428–0.833), 0.703 (95% CI: 0.575–0.833), 0.565 (95% CI: 0.391–0.718), and 0.655 (95% CI: 0.545–0.764), respectively.

**Figure 8:**
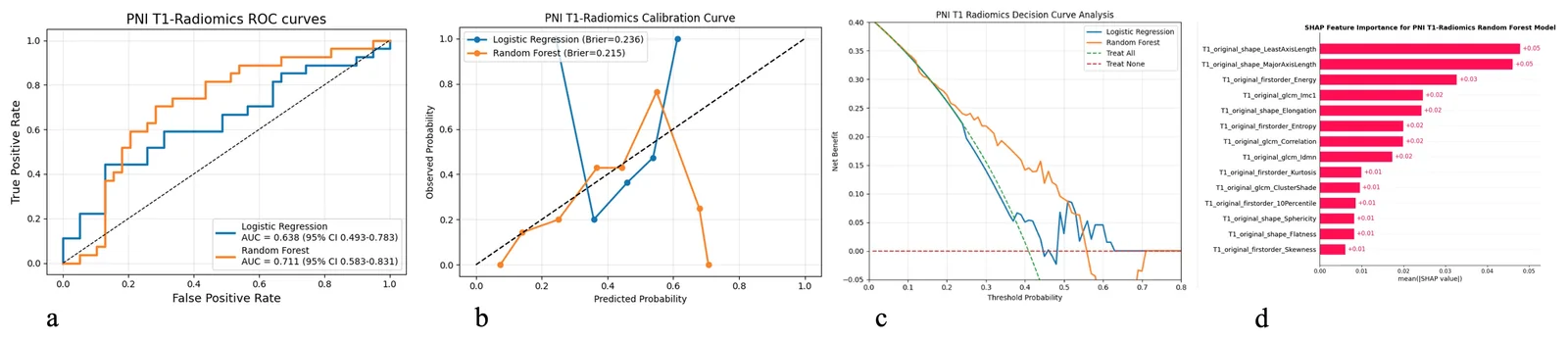
**(a–d)** ROC curve **(a)**, calibration curve **(b)**, decision curve analysis **(c)**, and important features **(d)** of the T1 radiomics model for predicting perineural invasion (PNI).

**Figure 8** depicts the ROC curves, calibration curves, DCA, and important features of the T1 radiomics predictive model for PNI. Corresponding analyses for the other models are presented in **Supplementary Figures 25–29**.

### Predictive modeling for metastatic nodal involvement

Metastatic nodes were seen in 105 patients (47.94%). The RF model outperformed the LR model in predicting metastatic nodes across both the combined and individual models. The T1c radiomics RF model demonstrated the highest overall predictive performance for metastatic nodes among all models, achieving mean AUC, accuracy, sensitivity, specificity, PPV, NPV, F1-score, and balanced accuracy of 0.746 (95% CI: 0.621–0.865), 0.742 (95% CI: (95% CI: 0.636–0.848), 0.686 (95% CI: 0.517–0.839), 0.794 (95% CI: 0.655–0.921), 0.758 (95% CI: 0.600–0.906), 0.729 (95% CI: 0.583–0.865), 0.717 (95% CI: 0.582–0.836), and 0.740 (95% CI: 0.634–0.845), respectively. Among all of the models, the radiology semantic features RF model demonstrated the highest mean AUC of 0.776 (95% CI: 0.656–0.883). The T2 RF model demonstrated the highest mean AUC among all radiomics-based models, with a value of 0.753 (95% CI: 0.629–0.862).

**Figure 9:**
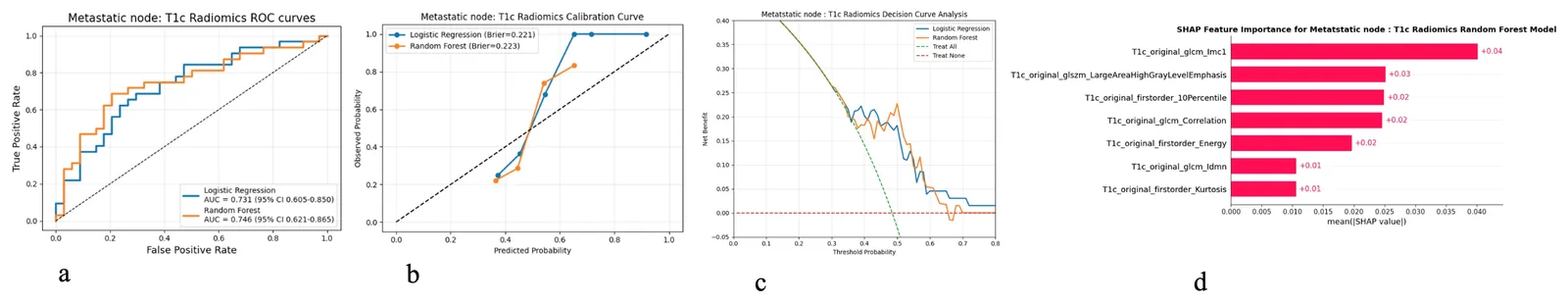
**(a–d)** ROC curve **(a)**, calibration curve **(b)**, decision curve analysis **(c)**, and important features **(d)** of the T1c radiomics model for predicting metastatic nodes.

The ROC curve, calibration curve, DCA, and important features of the T1c radiomics model for predicting metastatic lymph nodes are presented in **Figure 9**, while the corresponding results for the other models are provided in **Supplementary Figures 30–S34**.

### Predictive modeling for pENE

A total of 59 patients had pENE (26.94%). Among all of the models, only the radiology semantic features RF model and the T1c radiomics LR model demonstrated comparatively good overall predictive performance for pENE, with the radiology semantic features RF model outperforming the T1c radiomics LR model. The radiology semantic features RF model achieved a mean AUC of 0.792 (95% CI: 0.669–0.898), accuracy of 0.757 (95% CI: 0.652–0.864), sensitivity of 0.666 (95% CI: 0.429–0.883), specificity of 0.792 (95% CI: 0.667–0.891), PPV of 0.544 (95% CI: 0.333–0.750), NPV of 0.863 (95% CI: 0.756–0.957), F1-score of 0.593 (95% CI: 0.400–0.762), and balanced accuracy of 0.729 (95% CI: 0.598–0.858). The T1c radiomics LR model achieved a mean AUC of 0.754 (95% CI: 0.615–0.876), with corresponding accuracy, sensitivity, specificity, PPV, NPV, F1-score, and balanced accuracy of 0.696 (95% CI: 0.576–0.803), 0.891 (95% CI: 0.727–1.000), 0.622 (95% CI: 0.479–0.755), 0.470 (95% CI: 0.304–0.636), 0.938 (95% CI: 0.844–1.000), 0.610 (95% CI: 0.450–0.755), and 0.757 (95% CI: 0.651–0.850), respectively.

**Figure 10:**
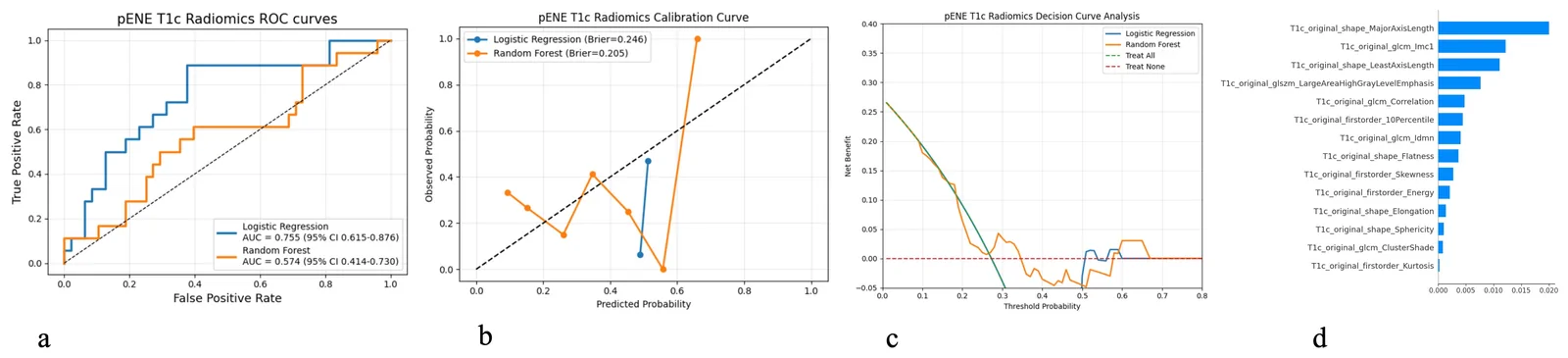
**(a–d)** ROC curve **(a)**, calibration curve **(b)**, decision curve analysis **(c)**, and important features **(d)** of the T1c radiomics model for predicting pathological extranodal extension (pENE).

The ROC curve, calibration curve, DCA, and important features of the T1c radiomics predictive model for pENE are depicted in **Figure 10**, while the corresponding results for the radiology semantic features model are provided in **Supplementary Figure 35**.

### Prognostication based on risk stratification for disease recurrence

**Figure 11:**
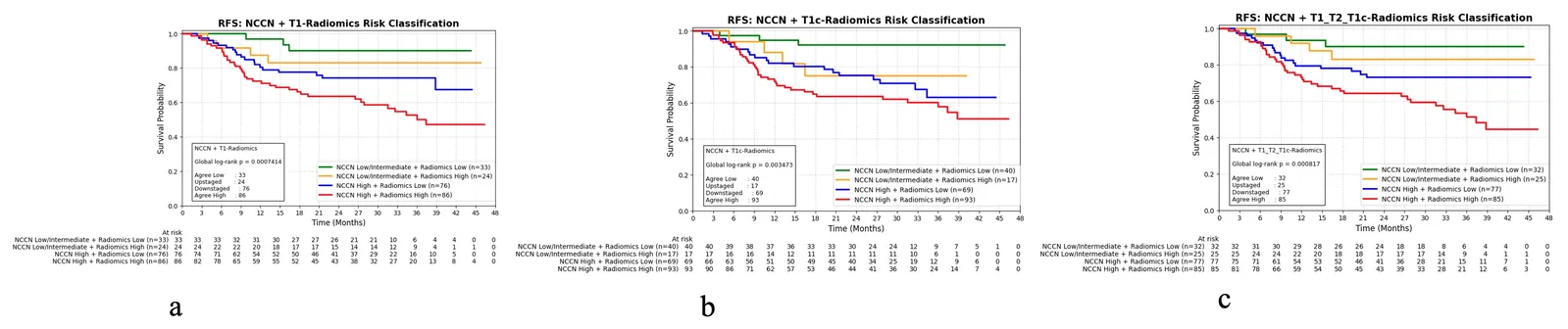
**(a–c)** Kaplan–Meier RFS curves demonstrating NCCN and radiomics-based risk stratification for the T1 radiomics model **(a)**, T1c radiomics model **(b)**, and T1 + T2 + T1c radiomics model **(c)**.

Clinico-pathologic low and intermediate risk groups based on NCCN recommendations were merged as there were fewer patients (7) in the intermediate risk group. The NCCN low + intermediate- and high-risk groups were correlated with the risk stratification (low and high) provided by the T1, T1c, and T1 + T2 + T1c radiomics models using median risk scores predicted by the Lasso–Cox model (**Tables 2**–**4**). KM RFS plots for NCCN and radiomics models are depicted in **Figure 11**.

**Table 2:** National comprehensive cancer network (NCCN) and T1 radiomics risk stratification for disease recurrence.

NCCN risk groups (low + intermediate versus high)	RFS T1 model low and high risk	*N*	*N* of events	Censored		Percentage of patients surviving 24 months
				*N*	Percent	
Low + intermediate	Low	33	3	30	90.91%	90.3%
	High	24	4	20	83.33%	83.1%
	Overall	57	7	50	87.72%	87.2%
High	Low	76	19	57	75%	74.3%
	High	86	38	48	55.81%	63.6%
	Overall	162	57	105	64.81%	68.6%
Overall	Overall	219	64	155	70.78%	73.6%

*N*, total number of patients; RFS, recurrence-free survival.

**Table 3:** National comprehensive cancer network (NCCN) and T1c radiomics risk stratification for disease recurrence.

NCCN risk groups (low + intermediate versus high)	RFS T1c model low and high risk	*N*	*N* of events	Censored		Percentage of patients surviving 24 months
				*N*	Percent	
Low + intermediate	Low	40	3	37	92.5%	92.3%
	High	17	4	13	76.47%	75.1%
	Overall	57	7	50	87.72%	87.2%
High	Low	69	20	49	71.01%	75.3%
	High	93	37	56	60.22%	63.8%
	Overall	162	57	105	64.81%	68.6%
Overall	Overall	219	64	155	70.78%	73.6%

*N*, total number of patients; RFS, recurrence-free survival.

**Table 4:** National comprehensive cancer network (NCCN) and T1 + T2 + T1c radiomics risk stratification for disease recurrence.

NCCN risk groups (low + intermediate versus high)	RFS_T1 + T2 + T1c_ model low and high risk	*N*	*N* of events	Censored		Percentage of patients surviving 24 months
				*N*	Percent	
Low + intermediate	Low	32	3	29	90.63%	90.3%
	High	25	4	21	84.0%	83.2%
	Overall	57	7	50	87.72%	87.2%
High	Low	77	19	58	75.32%	73.3%
	High	85	38	47	55.29%	64.4%
	Overall	162	57	105	64.81%	68.5%
Overall	Overall	219	64	155	70.78%	73.6%

*N*, total number of patients; RFS, recurrence-free survival.

The T1 + T2 + T1c radiomics model, as well as the T1 and T1c radiomics models, significantly (*p* < 0.05) identified low-risk patients within the NCCN high-risk group and high-risk patients within the NCCN low + intermediate-risk group. These findings suggest the potential utility of these models in guiding treatment de-escalation and escalation, respectively. However, these results are hypothesis-generating and require prospective multicenter validation before they can be incorporated into clinical decision-making.

**Supplementary Table 4** provides simplified interpretations of the important radiomics features for ease of reference. The radiomics quality score (RQS) of our study was 12 (attached as **Supplementary Material**) (30).

## Discussion

In our study, the radiomics combined model outperformed the individual models for RFS prediction. Additionally, the radiomics combined, T1 + T2 + T1c radiomics, T1c radiomics, and T1 radiomics models effectively stratified patients into high- and low-risk groups for recurrence, highlighting their potential to support risk-adapted management and identify patients who may benefit from a more aggressive treatment.

The radiomics combined model demonstrated non-inferior performance to the pathology model for predicting OS. Furthermore, the radiomics combined, T1c radiomics, and T1 radiomics models successfully stratified the patients into high- and low-risk groups for OS, highlighting their potential to inform risk-adapted management decisions. Among the individual radiomics models, the T1c radiomics model demonstrated the best performance for predicting both RFS and OS.

Lasso–Cox was found to be superior to DeepSurv for RFS and OS prediction across all of the models. The superior performance of the Lasso–Cox model compared to DeepSurv may be attributed to the relatively small sample size (*n* = 219), which may limit the ability of deep learning models to learn generalized survival representations without overfitting. In contrast, regularized linear models such as Lasso–Cox are more suitable for high-dimensional, low-sample-size data, as they impose sparsity and reduce variance.

The potential histopathological correlates of the top-weighted features of the radiomics-based models for increased recurrence risk and poor overall survival prediction are large-sized tumors with invasive margins, enhanced angiogenesis with increased microvessel density, uniform cellularity, and less cystic components.

The radiology semantic features RF model demonstrated the highest overall predictive performance for PNI among all of the evaluated models, followed by the T1 radiomics RF model. Potential histopathological correlates of the important radiomics features associated with an increased risk of PNI include greater depth of invasion and tumor thickness, high tumor cellularity, increased microscopic architectural heterogeneity, and an infiltrative pattern of invasion.

The T1c radiomics RF model demonstrated the best overall performance for predicting metastatic lymph nodes. The top radiomic features predictive of metastatic nodal disease may correspond to the histopathological characteristics of aggressive tumors, including extensive viable tumor with high cellularity, increased microvessel density, large confluent cohesive tumor nests, relative absence of necrosis, and greater tumor thickness and depth of invasion.

The radiology semantic features RF model demonstrated the highest predictive performance for pENE, followed by the T1c radiomics LR model. The key radiomic features associated with an increased risk of pENE may reflect underlying histopathological characteristics such as increased tumor cellularity, higher microvessel density, a predominance of viable tumor with minimal necrosis, an infiltrative pattern of tumor invasion, greater depth of invasion, and increased microscopic architectural heterogeneity.

Corti et al. (25) demonstrated a C index of 0.68 for predicting OS using MRI radiomics in locally advanced oral cavity carcinoma, which was similar to our T1c radiomics model which showed a value of 0.64 ± 0.06 SD. Although our radiomics combined model achieved a higher mean test C index (0.69 ± 0.07), the model developed by Corti et al. did not incorporate radiology semantic features (25). Bologna et al. (26) showed a similar OS prediction with a C index of 0.64 using MRI radiomics for head and neck cancer. In the study by Alfieri et al. (27), the C index for combined radiomics model was 0.74 for OS prediction and 0.76 for DFS prediction in head and neck cancers, whereas our study demonstrated C indices of 0.69 ± 0.07 SD and 0.70 ± 0.11 SD for OS and RFS prediction, respectively, using the Radiomics combined model.

Similar to the study by Tagliabue et al. (28), the T1c sequence was the best fit for OS prediction in tongue carcinoma. Our radiomics combined OS predictive model showed a better performance (mean test C index of 0.69 ± 0.07 SD) compared to that of clinical radiomics model (test C index of 0.61) in the study by Tagliabue et al. (28). Lan et al. (31) used radiomics as well as deep learning on MRI of primary tumor for predicting lymph node metastasis in oral cavity and oropharyngeal cancers and found AUC of 0.798 in the test cohort, whereas our study showed AUC of 0.753 (95% CI: 0.629–0.862) in the test cohort using T2 radiomics RF model. Mukherjee et al. (32) was the only study which used radiomics for the prediction of PNI, but on CT scan, and found AUC of 0.70 in the test cohort for predicting PNI, whereas our T2 radiomics LR model showed the highest mean AUC of 0.72 (95% CI: 0.578–0.841) in the test cohort.

The majority of radiomics-based studies in head and neck cancer have relied on CT imaging and often excluded radiology semantic features.

The RQS of our study was 12, which was higher than the mean of 9.40 documented in the literature (33).

The strength of our study lies in the fact that pre-treatment MRI images of tumors were manually segmented and eight predictive models (T1,T2, T1c radiomics individual and integrated models + radiology semantic features model + clinical model + pathology model + radiomics combined model incorporating all the models) were trained and tested for RFS and OS prediction as well as for prognostication based on risk stratification into high- and low-risk cohorts. To our knowledge, this is the first study to employ pre-treatment radiomics models to identify low-risk patients within the NCCN high-risk group and high-risk patients within the NCCN low + intermediate-risk group for prediction of recurrence. These findings suggest that these models may have potential utility in guiding treatment de-escalation and escalation, respectively. However, the results are hypothesis-generating and require prospective multicenter validation before clinical implementation. To our knowledge, this study is among the first to develop and evaluate radiomics-based predictive models for PNI using pre-treatment MRI of primary tumor.

Our study has several limitations. First, external validation using data from other institutions was not performed, which may limit the generalizability of the findings. Future studies should focus on multi-institutional validation using harmonized imaging protocols. Second, image intensity normalization was not applied prior to radiomics feature extraction. Future investigations may incorporate techniques such as ComBat harmonization and domain adaptation methods to mitigate scanner-related variability and enhance model generalizability. Third, although all tumor segmentations were verified and, where necessary, refined by an experienced radiologist, the numerical stability of radiomics features with respect to region-of-interest (ROI) perturbations was not evaluated. Fourth, resampling, gray-level discretization, and image harmonization were not performed, and these constitute further limitations of our study. In addition, interobserver and intraobserver segmentation reproducibility, respectively, were not evaluated, representing another limitation of our study.

## Conclusion

The radiomics combined, T1 + T2 + T1c radiomics, T1c radiomics, and T1 radiomics models effectively stratified patients into high- and low-risk groups for recurrence, while the radiomics combined, T1c radiomics, and T1 radiomics models similarly stratified patients according to OS risk. These findings highlight the potential of pre-treatment radiomics for risk-adapted management, enabling the identification of early-stage OTSCC patients who may benefit from treatment intensification and individualized surveillance strategies. Pre-treatment radiomics models identified low-risk patients within the NCCN high-risk group and high-risk patients within the NCCN low + intermediate-risk group for recurrence, highlighting their potential to support risk-adapted treatment de-escalation and escalation, respectively. This may also help to avoid overtreatment and reduce treatment-related toxicity in the radiomics-identified low-risk patients within the NCCN high-risk group. These hypothesis-generating findings warrant prospective multicenter validation before clinical implementation.

A high likelihood of metastatic lymph nodes predicted by the T1c radiomics model may support the decision to perform elective neck dissection on pre-treatment stage.

The T1 radiomics RF model may facilitate the preoperative identification of PNI on baseline MRI, which may assist in guiding decisions regarding adjuvant therapy following prospective validation. The T1c radiomics LR model has the potential to predict pENE from pre-treatment MRI, potentially enabling earlier risk stratification and informing decisions regarding adjuvant chemoradiotherapy following prospective validation.

## Data Availability

The original contributions presented in the study are included in the article/**Supplementary Material**. Further inquiries can be directed to the corresponding author.
