# Supplementary material for: MRI radiomics-based predictive modeling for risk stratification and prognostication in oral tongue carcinoma
Source: Front Oncol. 2026 Sep 3;16:1901400. doi: 10.3389/fonc.2026.1901400 (PMC13581926; doi:10.3389/fonc.2026.1901400)
Supplement: Supplementary file 2 [file DataSheet1.docx]

**Supplementary material**

**Table S1: MRI protocol parameters**

| **Sequences and plane** | **TR**  **GE/**  **Philips** | **TE**  **GE/**  **Philips** | **Flip angle**  **GE/**  **Philips** | **Receiver bandwidth**  **GE/**  **Philips** | **Slice thickness** | **Spacing** | **Field of view** |
| --- | --- | --- | --- | --- | --- | --- | --- |
| Coronal STIR | 6000/3977 | 42/35 | 160/90 | 62.5/394.6 | 4 mm | 1 mm | 230 mm |
| Coronal T1WI | 680/585 | 42/9 | 160/90 | 50/600 | 4 mm | 1 mm | 230 mm |
| Sagittal T2WI | 5500/3500 | 120/80 | 160/90 | 83.33/225 | 4 mm | 1 mm | 240 mm |
| Axial T1WI | 300-700/601 | 43/15 | 160/90 | 50/506 | 3 mm | 0.4 mm | 160 mm |
| Axial T2WI | 3000-8000/3000 | 120/80 | 160/90 | 50/506 | 3 mm | 0.4 mm | 160 mm |
| Axial T2WI | 3000-8000/3000 | 120/80 | 160/90 | 50/398.1 | 4 mm | 1 mm | 220 mm |
| DWI (b-values 0 and 800 s/mm^2^) | 6300/4800 | 85/70 |  | 250/18 | 4 mm | 1 mm | 300 mm |
| Axial T1 post contrast fat saturated | 300-700/520 | 30/8 | 160/90 | 42/524 | 3 mm | 0.4 mm | 160 mm |
| Coronal T1 post contrast fat saturated | 300-700/534 | 30/10 | 160/90 | 40/531 | 4 mm | 1 mm | 230 mm |
| Axial post contrast 3D FSPGR | 11-15/7.4 | 20/3.6 | 13/10 | 25/708.6 | ≤ 2 mm | 0.75 | 230 mm |
| STIR- Short tau inversion recovery, WI- Weighted Image, DWI- Diffusion Weighted Image, 3D FSPGR-Three Dimensional Fast Spoiled Gradient Echo (FSPGR) sequences | | | | | | | |

**Table S2: Feature selection stability**

| **Feature** | **Selected_in_Folds** | **Selection_Frequency** |
| --- | --- | --- |
| Adjuvant_chemoradiotherapy (CRT) | 5 | 1 |
| Extension of tumor till < 5 mm of hyoid bone | 5 | 1 |
| Tumor location from midline | 5 | 1 |
| Tumor_ulceration | 5 | 1 |
| pathological_N_category | 5 | 1 |
| Radiological_T_category | 5 | 1 |
| Perineural invasion (PNI) | 5 | 1 |
| T1c_original_firstorder_Kurtosis | 5 | 1 |
| T1_original_glcm_Idmn | 5 | 1 |
| T1_original_glcm_Correlation | 5 | 1 |
| T1c_original_glcm_Idmn | 5 | 1 |
| T1_original_firstorder_Skewness | 5 | 1 |
| No_adjuvant | 4 | 0.8 |
| T1c_original_glcm_ClusterShade | 4 | 0.8 |
| Distance_from_hyoid_bone | 3 | 0.6 |
| Peritumoral_edema | 3 | 0.6 |
| T1_original_shape_Flatness | 3 | 0.6 |
| Worst pattern of invasion (WPOI) | 3 | 0.6 |
| T1_original_shape_LeastAxisLength | 3 | 0.6 |
| T1_original_glszm_LargeAreaHighGrayLevelEmphasis | 3 | 0.6 |
| T2_original_glcm_ClusterShade | 3 | 0.6 |
| T1c_original_shape_Flatness | 2 | 0.4 |
| Imaging inner tumor margin | 2 | 0.4 |
| T1_original_shape_Sphericity | 2 | 0.4 |
| maximum_width_of_peritumoral_hyperenhancement_(mm) | 1 | 0.2 |
| T2_original_shape_Flatness | 1 | 0.2 |
| T1_original_glcm_Imc1 | 1 | 0.2 |
| T1c_original_glcm_Correlation | 1 | 0.2 |
| T1_original_firstorder_Energy | 1 | 0.2 |
| T2_original_firstorder_10Percentile | 1 | 0.2 |
| T1_original_glcm_ClusterShade | 1 | 0.2 |
| Frequency  1.0 = selected in all 5 folds  0.8 = selected in 4 out of 5 folds  0.6 = selected in 3 out of 5 folds  0.4 = selected in 2 out of 5 folds  0.2 = selected in 1 out of 5 folds | | |

**Table S3: Demographic and clinical data of the study population**

|  |  | **Early stage** | **Locally Advanced stage** | **Total n=219** |
| --- | --- | --- | --- | --- |
| **Mean Age** |  | 46 | 47.6 | 47 |
| **Gender** | Male | 65 (29.68%) | 114 (52.05%) | 179 (81.73%) |
|  | Female | 17 (7.76%) | 23 (10.50%) | 40(18.26%) |
| **pT categories** | pT1 | 33 (15.06%) | 9 (4.10%) | 42 (19.17%) |
|  | pT2 | 49 (22.37%) | 42 (19.17%) | 91 (41.55%) |
|  | pT3 | 0 | 68 (31.05%) | 68 (31.05%) |
|  | pT4a | 0 | 18 (8.21%) | 18 (8.21%) |
| **pN categories** | pN0 | 82 (37.44%) | 32 (14.61%) | 114 (52.05%) |
|  | pN1 | 0 | 26 (11.87%) | 26 (11.87%) |
|  | pN2a | 0 | 17 (7.76%) | 17 (7.76% |
|  | pN2b | 0 | 19 (8.67%) | 19 (8.67%) |
|  | pN2c | 0 | 1 (0.45%) | 1(0.45%) |
|  | pN3b | 0 | 42 (19.17%) | 42(19.17%) |
| **Adjuvant treatment** | RT | 26 (11.87%) | 72 (23.87%) | 98 (44.7%) |
|  | CRT | 0 | 58 (26.48%) | 58 (26.48%) |
|  | No Adjuvant | 56 (25.57%) | 7 (3.19%) | 63 (28.76%) |
| **Overall Stage** | I | 33 (15.06%) | 0 | 33 (15.06%) |
|  | II | 49 (22.37%) | 12 (5.47%) | 61 (27.85%) |
|  | III | 0 | 43 (17.80%) | 43 (19.63%) |
|  | IV | 0 | 82 (37.44%) | 82 (37.44%) |
| **Median RFS in months** |  | 30.7 months | 24.17 months | 27.7 months |
| pT: pathological primary tumor, pN: pathological regional node, RFS: Recurrence free survival, RT: Radiotherapy, CRT: Chemoradiotherapy | | | | |

Time-dependent AUC

Time_months Mean_AUC	STD_AUC N_Folds

0	12.0	0.569912	0.119358	5

1	24.0	0.604207	0.084680	5

2	36.0	0.598733	0.095921	5

a

b

Figure S1: Time-dependent AUC (a) and aggregated calibration curve across all folds (b) for Clinical model for Recurrence-free survival (RFS) prediction.

Time-dependent AUC

Time_months Mean_AUC	STD_AUC N_Folds

0	12.0	0.741622	0.046621	5

1	24.0	0.721849	0.054345	5

2	36.0	0.707144	0.053399	5

a

b

Figure S2: Time-dependent AUC (a) and aggregated calibration curve across all folds (b) for Pathology model for Recurrence-free survival (RFS) prediction.

Time-dependent AUC

Time_months Mean_AUC	STD_AUC N_Folds

0	12.0	0.651897	0.039487	5

1	24.0	0.628763	0.069158	5

2	36.0	0.584403	0.116022	5

a

b

Figure S3: Time-dependent AUC (a) and aggregated calibration curve across all folds (b) for Radiology semantic features model for Recurrence-free survival (RFS) prediction.

Time-dependent AUC

Time_months Mean_AUC	STD_AUC N_Folds

0	12.0	0.560098	0.062909	5

1	24.0	0.588843	0.103450	5

2	36.0	0.575712	0.036604	5

a

b

Figure S4: Time-dependent AUC (a) and aggregated calibration curve across all folds (b) for T1W Radiomics model for Recurrence-free survival (RFS) prediction.

Time-dependent AUC

Time_months Mean_AUC	STD_AUC N_Folds

0	12.0	0.519770	0.059371	5

1	24.0	0.556237	0.073300	5

2	36.0	0.578452	0.181922	5

a

b

Figure S5: Time-dependent AUC (a) and aggregated calibration curve across all folds (b) for T2W Radiomics model for Recurrence-free survival (RFS) prediction.

Time-dependent AUC

Time_months Mean_AUC	STD_AUC N_Folds

0	12.0	0.651921	0.064412	5

1	24.0	0.645251	0.049624	5

2	36.0	0.576201	0.060152	5

a

b

Figure S6: Time-dependent AUC (a) and aggregated calibration curve across all folds (b) for CE T1W Radiomics model for Recurrence-free survival (RFS) prediction.

Time-dependent AUC

Time_months Mean_AUC	STD_AUC N_Folds

0	12.0	0.600390	0.078484	5

1	24.0	0.622287	0.100117	5

2	36.0	0.594921	0.056937	5

a

b

Figure S7: Time-dependent AUC (a) and aggregated calibration curve across all folds (b) for T1W+T2W+ CE T1W Radiomics model for Recurrence-free survival (RFS) prediction.

Time-dependent AUC

Time_months Mean_AUC	STD_AUC N_Folds

0	12.0	0.746505	0.128664	5

1	24.0	0.749680	0.132816	5

2	36.0	0.732319	0.093949	5

a

b

Figure S8: Time-dependent AUC (a) and aggregated calibration curve across all folds (b) for Combined model including T1W, T2W and CE T1W Radiomics + Radiology semantic features + Pathology + Clinical models, for Recurrence-free survival (RFS) prediction.

Figure S9: Important Clinical features for RFS prediction.

Figure S10: Important Pathology features for RFS prediction.

Figure S11: Important Radiology semantic features for RFS prediction.

Figure S12: Important T2W Radiomics features for RFS prediction.

Time-dependent AUC

Time_months Mean_AUC	STD_AUC N_Folds

0	12.0	0.621437	0.106529	5

1	24.0	0.604048	0.060877	5

2	36.0	0.590355	0.056024	5

a

b

Figure S13: Time-dependent AUC (a) and aggregated calibration curve across all folds (b) for Clinical model for Overall survival (OS) prediction.

Time-dependent AUC

Time_months Mean_AUC	STD_AUC N_Folds

0	12.0	0.774251	0.103391	5

1	24.0	0.754555	0.094210	5

2	36.0	0.716098	0.081004	5

a

b

Figure S14: Time-dependent AUC (a) and aggregated calibration curve across all folds (b) for Pathology model for Overall survival (OS) prediction.

Time-dependent AUC

Time_months Mean_AUC	STD_AUC N_Folds

0	12.0	0.664688	0.094857	5

1	24.0	0.664793	0.048431	5

2	36.0	0.598299	0.075026	5

a

b

Figure S15: Time-dependent AUC (a) and aggregated calibration curve across all folds (b) for Radiology semantic features model for Overall survival (OS) prediction.

Time-dependent AUC

Time_months Mean_AUC	STD_AUC N_Folds

0	12.0	0.574441	0.088722	5

1	24.0	0.616149	0.062244	5

2	36.0	0.536008	0.125844	5

a

b

Figure S16: Time-dependent AUC (a) and aggregated calibration curve across all folds (b) for T1W Radiomics model for Overall survival (OS) prediction.

Time-dependent AUC

Time_months Mean_AUC	STD_AUC N_Folds

0	12.0	0.593927	0.071771	5

1	24.0	0.621482	0.021799	5

2	36.0	0.520631	0.106065	5

a

b

Figure S17: Time-dependent AUC (a) and aggregated calibration curve across all folds (b) for T2W Radiomics model for Overall survival (OS) prediction.

Time-dependent AUC

Time_months Mean_AUC	STD_AUC N_Folds

0	12.0	0.602752	0.078054	5

1	24.0	0.662837	0.073780	5

2	36.0	0.554030	0.112508	5

a

b

Figure S18: Time-dependent AUC (a) and aggregated calibration curve across all folds (b) for CE T1W Radiomics model for Overall survival (OS) prediction.

Time-dependent AUC

Time_months Mean_AUC	STD_AUC N_Folds

0	12.0	0.554004	0.082913	5

1	24.0	0.607942	0.071300	5

2	36.0	0.535406	0.133591	5

a

b

Figure S19: Time-dependent AUC (a) and aggregated calibration curve across all folds (b) for T1W+T2W+ CE T1W Radiomics model for Overall survival (OS) prediction.

Time-dependent AUC

Time_months Mean_AUC	STD_AUC N_Folds

0	12.0	0.745711	0.095839	5

1	24.0	0.724378	0.075043	5

2	36.0	0.671574	0.106909	5

a

b

Figure S20: Time-dependent AUC (a) and aggregated calibration curve across all folds (b) for Combined model including T1W, T2W and CE T1W Radiomics + Radiology semantic features + Pathology + Clinical models, for Overall survival (OS) prediction.

Figure S21: Important Clinical features for OS prediction.

Figure S22: Important Pathology features for OS prediction.

Figure S23: Important Radiology semantic features for OS prediction.

Figure S24: Important T2W Radiomics features for OS prediction.

a

c

b

d

Figure S25 (a-d): ROC curve (a), calibration curve (b), decision curve analysis (c) and important features (d) of the Radiology semantic features model for predicting perineural invasion (PNI).

a

b

c

d

Figure S26 (a-d): ROC curve (a), calibration curve (b), decision curve analysis (c) and important features (d) for T2 Radiomics model for predicting perineural invasion (PNI).

a

b

c

d

Figure S27 (a-d): ROC curve (a), calibration curve (b), decision curve analysis (c) and important features (d) for T1c Radiomics model for predicting perineural invasion (PNI).

a

b

c

d

Figure S28 (a-d): ROC curve (a), calibration curve (b), decision curve analysis (c) and important features (d) for T1+T2+T1c Radiomics model for predicting perineural invasion (PNI).

a

b

c

d

Figure S29 (a-d): ROC curve (a), calibration curve (b), decision curve analysis (c) and important features (d) of the Combined T1+T2+T1c Radiomics and Radiology semantic features model for predicting perineural invasion (PNI).

a

b

c

d

Figure S30 (a-d): ROC curve (a), calibration curve (b), decision curve analysis (c) and important features (d) of the Radiology semantic features model for predicting metastatic nodes.

a

b

c

d

Figure S31 (a-d): ROC curve (a), calibration curve (b), decision curve analysis (c) and important features (d) of the T1 Radiomics model for predicting metastatic nodes.

a

b

c

d

Figure S32 (a-d): ROC curve (a), calibration curve (b), decision curve analysis (c) and important features (d) of the T2 Radiomics model for predicting metastatic nodes.

a

b

c

d

Figure S33 (a-d): ROC curve (a), calibration curve (b), decision curve analysis (c) and important features (d) of the T1+T2+T1c Radiomics model for predicting metastatic nodes.

b

a

c

d

Figure S34 (a-d): ROC curve (a), calibration curve (b), decision curve analysis (c) and important features (d) of the combined T1+T2+T1c Radiomics and Radiology semantic features model for predicting metastatic nodes.

a

b

c

d

Figure S35 (a-d): ROC curve (a), calibration curve (b), decision curve analysis (c) and important features (d) of the Radiology semantic features model for predicting pathological extranodal extension (pENE).

**Table S4: Important Radiomics features and their simplified interpretation**

| **Important Radiomics features** | **Simplified interpretation** |
| --- | --- |
| original_glcm_Correlation | Extent of similarity between adjacent voxels |
| original_firstorder_Skewness | Intensity asymmetry |
| original_firstorder_Kurtosis | Enhancement concentration |
| original_shape_LeastAxisLength | Smallest tumor dimension |
| original_ shape_Flatness | Tumor flatness |
| original_ glcm_Idmn (inverse difference moment normalised) | Texture uniformity |
| original_ glcm_ClusterShade | Tumor texture (intensity pattern) asymmetry |
| original_shape_Sphericity | Tumor roundness |
| original_glszm_LargeAreaHighGrayLevelEmphasis | Extent of large high intensity regions within the tumor |
| original_firstorder_10Percentile | Intensity below which darkest 10% of the tumor lies |
| original_shape_MajorAxisLength | Largest tumor dimension |
| original_firstorder_Energy | Overall signal intensity within the tumor |
| original_firstorder_Entropy | Extent of heterogeneity in voxel intensities |
| original_firstorder_Minimum | Extent of lowest voxel intensity value within the tumor |
| original_glcm_Imc1 (Informational Measure of Correlation 1) | Texture complexity within the tumor |
| original_shape_Elongation | Extent of tumor elongation |
